# Banting memorial lecture 2025: Aligning clinical practice, policy and research

**DOI:** 10.1111/dme.70245

**Published:** 2026-02-09

**Authors:** Jonathan Valabhji

**Affiliations:** ^1^ Department of Metabolism, Digestion and Reproduction, Faculty of Medicine Chelsea and Westminster Hospital Campus, Imperial College London London UK

**Keywords:** COVID‐19, type 2 diabetes prevention, type 2 diabetes remission, diabetic foot disease, implementation, lifestyle interventions, multimorbidity / multiple long‐term conditions, real‐world data

## Abstract

National clinical leadership, on a background of clinical practice and clinical research, provides unique perspectives. I have focused the Banting Memorial Lecture 2025 on the implementation of national programmes across England since 2013, for which, along with colleagues at NHS England, I successfully made the case for investment, led the implementation of interventions applied at scale across the country and used routinely collected healthcare data to demonstrate clinical effectiveness in the real world. Through specific examples of implemented programmes, including the NHS Diabetes Prevention Programme and the NHS Type 2 Diabetes Path to Remission Programme, I highlight important fundamental principles when making the case for, and implementing, national policy. First, ensure granular data collection to support evaluation and exploit data linkages to harness the power of real‐world datasets. Second, where good evidence exists, implement evidence‐based policy; where good evidence does not exist but political pressures to implement are being brought to bear, pilot and evaluate. Third, when the opportunity arises, rapidly translate new high‐quality evidence into policy and practice. And fourth, support and protect the workload of healthcare professionals, particularly of those working in primary care. Then, through an epidemiological lens, I highlight: how the COVID‐19 pandemic further unlocked the potential of national routinely collected electronic healthcare datasets; how, through application of these datasets, it has been possible to demonstrate improvements in diabetes complications and mortality through routine care delivery; and how it has been possible to demonstrate the next epidemiological transition in the global diabetes epidemic to multimorbidity/multiple long‐term conditions.

## 1 INTRODUCTION

My career in Diabetes has been an unusual one. Following around 11 years as a full‐time NHS Consultant at St Mary's Hospital in West London, part of Imperial College Healthcare NHS Trust from 2008, I spent just over a decade as the National Clinical Director for Diabetes and Obesity at NHS England. I stepped down as National Clinical Director in September 2023 to take up my current post as Clinical Chair in Medicine at Imperial College London based at Chelsea and Westminster Hospital. The combination of these three career chapters has given me somewhat unique perspectives. To deliver the Banting Memorial Lecture is a great honour, and it has presented an opportunity to align thoughts around the three associated facets of my career: clinical practice, policy and research.

I first attended a Diabetes UK Professional Conference 30 years ago as a junior registrar trainee in 1995. The organisation was then called the British Diabetes Association. A highlight of one of my first meetings was listening to Michael Bliss, who, in his book ‘The Discovery of Insulin’, related a provocative account of Banting's work, with colleagues Best, Collip and Macleod, to demonstrate that a protein extractable from the pancreas was the missing factor in people with Type 1 diabetes and that replacing it would lower blood glucose.^1^ They purified the protein, creating a life‐saving treatment for what had been a universally fatal disease. Framed by this greatest of scientific achievements, I hope I can relate some more modest and more recent attempts to improve the lives of people living with diabetes in England and attempts to prevent or delay the onset of type 2 diabetes in those at risk.

As healthcare professionals we have the great privilege of supporting people one at a time through the one‐to‐one doctor‐patient relationship. However, to combine this experience with the population‐level perspectives afforded by national clinical leadership is a greater privilege still. I focus this Banting Memorial Lecture on the implementation of national programmes of work across England since 2013, for which, along with colleagues at NHS England, I successfully made the case for investment, led the implementation of interventions applied at scale across the country and used routinely collected healthcare data to demonstrate clinical effectiveness in the real world.

My background in clinical research, as well as years in clinical practice, has provided insight into the need to adhere to some fundamental principles when making the case for, and implementing, national policy. First, ensure granular data collection to support evaluation and exploit data linkages to harness the power of real‐world datasets. Second, where good evidence exists, implement evidence‐based policy; where good evidence does not exist but political pressures to implement are being brought to bear, pilot and evaluate. Third, when the opportunity arises, rapidly translate high‐quality evidence outputs into policy and practice. And fourth, support and protect the workload of healthcare professionals, in particular of primary care, and develop workforce resilience through incorporation of delivery by non‐healthcare professionals where appropriate and possible, particularly for the delivery of lifestyle interventions. Crucial in the effective development and delivery of these workstreams was an effective partnership between the arms‐length bodies NHS England and Public Health England (latterly the Office for Health Improvement and Disparities at the Department of Health and Social Care), and our third sector colleagues at Diabetes UK.

The decade as National Clinical Director was punctuated by funding opportunities, including post‐general election UK Treasury Comprehensive Spending Reviews, which facilitated funding of policies with the potential to improve population health. Table 1 lists the national programmes implemented over the period for which I was National Clinical Director. I will focus on a few, and then, through an epidemiological lens, highlight: how the COVID‐19 pandemic further unlocked the potential of national routinely collected healthcare electronic datasets; how, through the application of these datasets, it has been possible to demonstrate improvements in diabetes complications and mortality through routine care delivery; and how it has been possible to demonstrate the next epidemiological transition in the global diabetes epidemic to multimorbidity/multiple long‐term conditions.

**TABLE 1:** NHS England Diabetes and Obesity Implementation Programmes 2013–2023.

Lifestyle interventions
The NHS Diabetes Prevention Programme
The NHS Type 2 Diabetes Path to Remission Programme
The NHS Digital Weight Management Programme
Transformation programmes supported initially through funding provided via the 2015 Comprehensive Spending Review
Supporting diabetes care process delivery and treatment target achievement nationally, then later focusing on routine care delivery recovery following the COVID‐19 pandemic
Supporting diabetes self‐management through digitally and face‐to‐face delivered structured education/empowerment programmes
Improving access to multidisciplinary diabetes foot care
Improving access to diabetes inpatient specialist nurse care
Programmes that followed publication of the NHS Long‐Term Plan in 2019
Implementing technology including continuous glucose monitoring in Type 1 diabetes and in pregnancy with diabetes and a pilot programme for hybrid closed loop systems/artificial pancreas
Improving treatment and care for children and young people with diabetes
Improving identification and management of monogenic diabetes
Multidisciplinary support for those with Type 1 diabetes and disordered eating
Establishment of the National Obesity Audit in April 2022
Improving treatment and care for those with Young‐Onset Type 2 Diabetes (prevalent Type 2 diabetes up to age 40 years)

## 2 DIABETIC FOOT DISEASE

Where good evidence exists, implement evidence‐based policy.

I was first appointed at St Mary's Hospital in 2002 as a Consultant in General Medicine with a Special Interest in Diabetes and Endocrinology. Mike Edmonds at Kings College Hospital had demonstrated in the 1980s that establishment of a multidisciplinary diabetes foot clinic was associated with a halving of major amputation incidence.^2^ One of my first actions around service improvement was to establish a multidisciplinary diabetes footcare team and service at St Mary's in 2002. Over the years this expanded from a once monthly combined clinic with diabetologist, podiatrist, orthotist and vascular surgeon, to daily clinics involving a large team of podiatrists, multiple rooms in a dedicated footcare outpatient department, with additional input from orthopaedic surgeon, microbiologist and neurologist as necessary. The clinic formed the basis for my clinical research in diabetic foot disease, with subsequent published outputs based on clinical observations, including for Charcot neuroarthropathy,^3^, ^4^ conservative management of gangrenous toes^5^ and diabetic foot ulceration with associated bone infection.^6^ We were able to demonstrate particularly low major amputation rates following establishment of the service, rates that could be benchmarked internationally.^7^

One of the funding opportunities that arose earlier in my time as National Clinical Director was through the UK Treasury Comprehensive Spending Review that followed the 2015 UK General Election. Through this I successfully made the case for additional funding to support better access to such multidisciplinary footcare services across England. This involved establishing services at hospitals where they did not previously exist and expanding capacity if necessary where they did already exist. This amounted to an overall additional investment of around £50million over 8 years. Recent data (Office for Health Improvement & Disparities 2023) demonstrated English major amputation incidence is now lower than in most other countries with significant reduction since 2012 (7.7 vs. 9.1 per year per 10,000 people with diabetes).

However, to many of us in the field it has become increasingly clear that premature mortality for this patient cohort is a much greater risk than lower‐limb amputation.^8^ I therefore latterly focused on the high mortality in this patient group and established a national pilot service implementing 12‐lead ECGs on presentation with foot ulceration, with a resulting signal of mortality benefit.^9^

Working with Kamlesh Khunti at Leicester University, and with National Institute for Health and Care Research Programme Grant funding, we are developing and evaluating a multifactorial intervention to improve cardiovascular outcomes in adults with type 2 diabetes and current or previous diabetic foot ulcers (MiFoot).^10^

## 3 THE NHS DIABETES PREVENTION PROGRAMME

Where good evidence exists, implement evidence‐based policy.

Ensure granular data collection to support evaluation and exploit data linkages to harness the power of real‐world datasets.

In 2014 I argued successfully for inclusion in the NHS future strategy document called ‘The Five‐Year Forward View’ an ambition for England to become the first country to implement at scale a national evidence‐based type 2 diabetes prevention programme.^11^ The NHS Health Check had been implemented in 2009 to assess people ages 40–75 years for risk factors for heart disease, stroke, diabetes and kidney disease and was already identifying swathes of the population with prediabetes,^12^ described by the National Institute of Health and Care Excellence as non‐diabetic hyperglycaemia (NDH).^13^ However, there was at that point very little service provision for individuals with NDH to beneficially modify their higher risk of developing type 2 diabetes.

A number of high‐quality well‐conducted randomised controlled trials on different continents had already demonstrated the ability to prevent or delay onset of type 2 diabetes in those with impaired glucose tolerance through intensive lifestyle interventions.^14^, ^15^, ^16^, ^17^, ^18^ A more recent UK study demonstrated similar reductions in type 2 diabetes incidence in those for whom risk status had been defined by HbA1c and fasting glucose levels, similar to the criteria outlined in NICE guidelines.^13^, ^19^

Following the 2015 General Election and the subsequent Spending Review, the Five‐Year Forward View and its ambition to implement an NHS Diabetes Prevention Programme (NHS DPP) were supported and funded. I established and chaired an expert advisory group which developed a national service specification for the intensive lifestyle intervention based on best evidence at that time. The service specification was used to run a national procurement of service providers capable of delivering the content of the service specification anywhere in England. Each local area in England was then able to appoint a provider from the national framework that best met local population needs. By the end of the first wave of national roll‐out (financial year April 2016 to March 2017), 51% geographical coverage of England was achieved; by the end of the second wave (April 2017 to March 2018), 75%; and by Summer 2018, England became the first country internationally to achieve full geographical coverage with such a programme. Granular data collection via a dedicated programme dataset which providers were obliged contractually to populate regularly for every person referred, linked to the National Diabetes Audit, supported evaluation. Near real‐time data collection allowed our programme expert advisory group to analyse and determine early signals that supported iterative programme development.^20^, ^21^

## 4 THE DIGITAL DIABETES PREVENTION PROGRAMME PILOT

Where good evidence does not exist but political pressures to implement are being brought to bear, pilot and evaluate.

The evidence for digital delivery of intensive lifestyle interventions to prevent type 2 diabetes was immature when we first procured providers of the NHS DPP to the national framework, and so, all providers initially delivered group‐based face‐to‐face approaches. The lack of digital delivery drew criticism in some political circles, and so, we undertook to pilot and evaluate digital provision.^22^ In terms of weight loss and HbA1c reduction trajectories, we were able to demonstrate that digital modes of delivery were at least as good.^23^, ^24^, ^25^ Fortuitously, results were available just before onset of the COVID‐19 pandemic, so that we could confidently switch to digital and remote delivery when group‐based face‐to‐face delivery was no longer possible.^26^ Post‐pandemic, the NHS DPP now offers participants the choice of digital or group‐based face‐to‐face delivery.

The National Institute for Health and Care Research (NIHR) commissioned an independent evaluation of the programme: ‘Diabetes Prevention—Long‐Term Multimethod Assessment programme (DIPLOMA)’. Policy making often attracts criticism, but an NIHR‐funded independent evaluation supported buy‐in from the clinical and academic communities, as well as supporting effective evolution of the programme through methodological expertise that the independent team brought and through their insights into the importance of behaviour change techniques as well as the importance of maintaining fidelity to initial behaviour change intentions over the course of translation into routine programme delivery. The DIPLOMA team have since demonstrated reduced type 2 diabetes incidence among programme participants^27^ and at English population level associated with programme implementation,^28^ cost effectiveness at 1 year^29^ and cost savings by year 9.^30^

Over 2 million people with NDH have now been referred into the NHS DPP. Recent retrospective analyses have raised the possibility that the intensive lifestyle intervention may be associated with reduced subsequent incidence of other and multiple long‐term conditions.^31^

## 5 THE NHS TYPE 2 DIABETES PATH TO REMISSION PROGRAMME

Rapidly translate high‐quality evidence outputs into policy and practice.

Support and protect the workload of healthcare professionals and develop workforce resilience through incorporation of delivery by non‐healthcare professionals where appropriate and possible.

In 2018 Roy Taylor and Mike Lean published the DIRECT trial, turning opinion on its head with regard what was thought to be the inexorably progressive nature of the hyperglycaemia of type 2 diabetes, to suggest that through fairy profound weight loss, disease remission could be achieved.^32^ However, delivery of the intervention, which involved a total meal replacement diet over 3 months followed by ongoing behavioural support to complete a 12‐month intervention, was by healthcare professionals in general practice, which would not be operationalizable at scale. A few months later Susan Jebb and Paul Aveyard in Oxford published the DROPLET trial, demonstrating that delivery of a very similar intervention in a trial setting could be undertaken by trained non‐healthcare professionals.^33^

I argued successfully for inclusion in the next NHS strategy document, published in January 2019 and called the ‘NHS Long Term Plan’,^34^ an ambition to initially pilot the DIRECT intervention, with delivery by non‐healthcare professionals. The pilot was successful, with associated weight loss highly encouraging, permitting subsequent implementation at scale nationally across England. The same approach was adopted: I chaired an expert advisory group that developed the service specification, with the service specification then used for national procurement with appointment of providers to a national framework, and national geographical coverage was achieved by April 2024. Using data from the first 1740 participants who had time to finish the programme before the end of 2022, we were able to demonstrate a type 2 diabetes remission rate of around 30% at 12 months.^35^

## 6 THE EFFICACY‐TO‐EFFECTIVENESS GAP

Ensure granular data collection to support evaluation and exploit data linkages to harness the power of real‐world datasets.

Of note, our remission rate at 12 months was less than that reported in the DIRECT trail, 30% vs. 46%. Effect sizes can be attenuated when pragmatic interventional implementation within the real world is compared with interventional testing in randomised controlled trial (RCT) settings. RCTs involve ideal circumstances and ideal participants, often highly selected from much larger potential participant populations, to derive maximum interventional efficacy. To accurately assess clinical and cost effectiveness, impact on population health, affordability and value for health systems and the populations that they serve, policy makers need some indication as to the effectiveness of interventions when implemented in the real world.^36^ This highlights the importance of evaluating and publishing interventional implementation in the real world.

The European Commission Horizon Europe 2022 Programme has funded our ‘Real‐World Evidence for Decisions in Diabetes (REDDIE)’ programme of work.^37^ This will include assessment of efficacy versus effectiveness gaps—it will use data from four large national registries, including the National Diabetes Audit in England, to derive trial emulation cohorts in whom the effectiveness of therapeutic interventions in the real world can be compared with the efficacy demonstrated when the same therapeutic interventions were tested in RCT settings.

## 7 ROUTINE DIABETES CARE, REAL‐WORLD DATA AND CLINICAL OUTCOMES

Ensure granular data collection to support evaluation and exploit data linkages to harness the power of real‐world datasets.

The diabetes community in England is indebted to the late Bob Young, a close friend and colleague, who through great insight and a drive to improve the quality of diabetes care delivery, established the National Diabetes Audit (NDA) in 2003. Since around 2016, the NDA has constituted an almost fully comprehensive register of all those with a coded diagnosis of diabetes in England, and from 2017 has also collated data on all those with a coded diagnosis of NDH.^38^ As well as supporting evaluation of the NHS Diabetes Prevention Programme, this has allowed us to track year‐on‐year for people living with diabetes delivery of the eight care processes, achievement against the three treatment targets (for HbA1c, blood pressure and lipids); and changes in complication and mortality rates by diabetes type.^39^, ^40^ Delivery of care processes and achievement of treatment targets have been associated with lower mortality,^41^, ^42^ lower hospital admissions,^43^ lower incident lower‐limb amputations^44^ and lower eye complications.^45^

Using NDA data, we were able to demonstrate that reductions in routine diabetes care during the COVID‐19 pandemic were associated with increased non‐COVID mortality,^46^ leading to a £37 million national investment in diabetes care recovery.

## 8 THE POWER OF NATIONAL DATASET LINKAGE: DIABETES AND COVID‐19

Early 2020 was a concerning time for us within the Diabetes Programme Team at NHS England, as early reports from China suggested an excess of people with diabetes among those dying from COVID‐19. Early reports were not able to differentiate the effects of diabetes status from the effects of increasing age. We realised that we were ideally placed to disentangle some of those early unknowns through national dataset linkages within the NHS England and NHS Digital data environments. We linked the National Diabetes Audit with the Master Patient Index that includes all those registered with a GP in England, with the Bridges to Health National Segmentation dataset and with Office for National Statistics mortality data. For the first time internationally, we were able to calculate the increased absolute and relative risks of COVID‐19‐related mortality in people with diabetes by type.^47^, ^48^ This evidence informed UK vaccine prioritisation policy when vaccines became available later that same year.

## 9 INCREASED LONGEVITY, DIVERSIFICATION OF THE CAUSES OF MORBIDITY AND MORTALITY IN DIABETES AND MULTIMORBIDITY/MULTIPLE LONG‐TERM CONDITIONS (MLTC)

From the turn of the millennium, we have seen in people with diabetes impressive reductions in both mortality from cardiovascular disease and hospitalisations from cardiovascular disease,^49^, ^50^ although greater gains were made prior to the financial crash in 2008.^39^, ^40^ This has resulted in increased longevity in people with diabetes, as well as a diversification of the causes of morbidity and mortality, with much greater contributions from non‐traditionally associated conditions including cancer and infections. Coupled with the younger age of onset of type 2 diabetes driven by our expanding waistlines societally, we are now seeing many more people living with diabetes and other multiple long‐term conditions, representing what we have described as the next epidemiological transition in the global diabetes epidemic.^51^

In my latter years as National Clinical Director for Diabetes and Obesity, I established a MLTC work stream within the programme, focussed primarily on data analytics using the same configuration of linked datasets that we employed during the COVID‐19 pandemic. After standing down as National Clinical Director in September 2023, I retained national leadership of this MLTC work stream, with sequential titles of ‘National Clinical Lead’ and then ‘National Specialty Advisor’ for MLTC. Whole‐population analyses have allowed us to define MLTC prevalence in England,^52^ and to quantify the years lived with and lost to diabetes‐associated MLTC.^53^

The recent suggested association between completion of the NHS Diabetes Prevention programme and reduced incidence of other and multiple long‐term conditions, aside from type 2 diabetes, is supporting the establishment of a pilot to evaluate the effectiveness of access to a lifestyle intervention (a tailored version of the NHS DPP) in people living with hypertension in reducing the incidence of long‐term conditions when compared to usual care. This will be run as a stepped‐wedge cluster randomised controlled trial in 216 GP practices (clusters) within eight healthcare administrative areas in England (called Integrated Care Systems), over 4 years.^54^

## 10 CONCLUSION

The different perspectives born out of the experiences of clinical practice, research and policy‐making led over the course of more than a decade to many national programmes of work that I hope have improved the care delivered to people living with diabetes, and I hope have also reduced the chances of many people from developing type 2 diabetes in the first place. The rapid translation of high‐quality research into national policy and practice and, using real‐world data, the establishment of models for evaluation to assess translational impact have proved to be achievable goals.

## FUNDING INFORMATION

None

## CONFLICTS OF INTEREST STATEMENT

Nothing to declare

.
